# miRNA as a New Regulatory Mechanism of Estrogen Vascular Action

**DOI:** 10.3390/ijms19020473

**Published:** 2018-02-06

**Authors:** Daniel Pérez-Cremades, Ana Mompeón, Xavier Vidal-Gómez, Carlos Hermenegildo, Susana Novella

**Affiliations:** 1 Department of Physiology, Faculty of Medicine and Dentistry, University of Valencia, 46010 Valencia, Spain; daniel.perez@uv.es (D.P.-C.); ana.mompeon@uv.es (A.M.); xavier.vidal@uv.es (X.V.-G.); carlos.hermenegildo@uv.es (C.H.); 2 INCLIVA Biomedical Research Institute, 46010 Valencia, Spain

**Keywords:** miRNA, estradiol, estrogen receptors, epigenetic regulation, endothelial cells

## Abstract

The beneficial effects of estrogen on the cardiovascular system have been reported extensively. In fact, the incidence of cardiovascular diseases in women is lower than in age-matched men during their fertile stage of life, a benefit that disappears after menopause. These sex-related differences point to sexual hormones, mainly estrogen, as possible cardiovascular protective factors. The regulation of vascular function by estrogen is mainly related to the maintenance of normal endothelial function and is mediated by both direct and indirect gene transcription through the activity of specific estrogen receptors. Some of these mechanisms are known, but many remain to be elucidated. In recent years, microRNAs have been established as non-coding RNAs that regulate the expression of a high percentage of protein-coding genes in mammals and are related to the correct function of human physiology. Moreover, within the cardiovascular system, miRNAs have been related to physiological and pathological conditions. In this review, we address what is known about the role of estrogen-regulated miRNAs and their emerging involvement in vascular biology.

## 1. Introduction

Estrogen is involved in many physiological processes, including sexual development and reproduction, regulation of skeletal homeostasis, lipid and carbohydrate metabolism, electrolyte balance, central nervous system function (including cognition and behavior), and cardiovascular system regulation [1,2]. In addition to its physiological relevance, the effects of estrogen (or its absence) on target tissues are related to the development of numerous diseases, which include various types of well-known hormone-dependent cancers including breast, ovarian, endometrial, and prostate cancer, among others. However, estrogen is also implicated in the progression of osteoporosis, neurodegenerative diseases, metabolic disorders (insulin resistance and obesity), autoimmune diseases (lupus erythematosus, multiple sclerosis, and rheumatoid arthritis), endometriosis, and cardiovascular diseases [3].

Sex differences in cardiovascular diseases have been extensively reported [4], suggesting that sex hormones have an important influence on the cardiovascular system. Indeed, statistical data have shown that women develop cardiovascular disease 7–10 years later than men [5]. In addition, epidemiological studies have provided evidence that cardi ovascular diseases are more frequent in men than in premenopausal women of the same age. However, during the fifth decade of a woman’s life, the decrease in estrogen levels that occurs in menopause is accompanied by an increase in the incidence of cardiovascular diseases [6,7], suggesting that estrogen plays a beneficial role in cardiovascular system. 

Based on the beneficial role of estrogen, hormonal replacement therapies (HRT) have been used in postmenopausal women with controversial findings [8,9]. The current consensus on HRT indicates that the vascular protective effects of estrogen depend on the onset of treatment after menopause, which has been recently reviewed in depth elsewhere [10]. The phenomenon, referred to as the “timing hypothesis”, postulates that the beneficial effects of hormonal replacement in the prevention of cardiovascular disease may occur only when hormonal supplementation is initiated before the detrimental effects that aging has on the cardiovascular system have become established [11]. In this regard, it has been reported that age moderates the vasodilatory [12] and anti-inflammatory [13] effects that estrogen have on vascular tissue in postmenopausal women.

Estrogen can modulate the cardiovascular system by acting directly on vascular cells or indirectly by systemic effects. Endothelial cells, vascular smooth muscle cells (VSMCs), and cardiomyocytes are estrogen targets because they express estrogen receptors (ER) [14]. In addition, ER expression described in monocytes, macrophages, and dendritic cells suggests that modulation of inflammatory processes, a key event in the initiation and development of cardiovascular diseases, may also be estrogen-dependent [15,16].

ERs function through two predominant mechanisms. In the “classical” mechanism, estrogen diffuses into the cell and binds the ERs, creating a complex that then binds to specific DNA motifs called estrogen response elements (EREs) in the promoter region of estrogen-responsive genes [17]. Classical mechanisms are mediated by two main ER isoforms, ERα and ERβ, which form homo- or heterodimers before binding to EREs, and which induce changes in gene expression. Several studies have provided evidence that ERα and ERβ have different physiological functions [18]. Indeed, these subtypes can have opposing gene-expression regulatory effects [19,20] and also have redundant mediatory roles [21,22]. In addition, estrogen signaling is selectively regulated by the relative balance between ERα and ERβ expression in target organs [23], although studies using ERα and ERβ knockout mice revealed that the beneficial effects estrogen has on the vascular system are mainly mediated by ERα [24,25].

Besides their classic genomic action, ERs can also trigger faster responses (in minutes) through plasma membrane receptors. Indeed, ERα and ERβ are present in plasma membranes and other cytoplasmic organelles such as mitochondria and endoplasmic reticulum membranes [26]. In addition, the recently described G protein-coupled ER (GPER) is also expressed in vascular tissues [27]. Indeed, many of the beneficial effects of estrogen seen in human and animal models, such as reduced myocardial pro-inflammatory cytokine expression, inhibition of VSMC proliferation, and nitric oxide (NO)-dependent vasodilation [28], have been recently attributed to the presence of GPER in the cardiovascular system.

## 2. Role of Estrogen in Vascular Physiology

As described above, vascular tissues are targets for sex hormones because specific receptors are expressed in both endothelial cells and VSMCs [14] and clinical and experimental data have demonstrated that estrogen has beneficial effects at the cardiovascular level [29,30]. In general, these protective effects have been attributed to their role in increasing arterial vasodilation and inhibiting inflammatory processes, which, in turn, prevent the development of atherosclerosis [6]. Moreover, estrogen can also indirectly influence plaque progression by modulating systemic lipid metabolism [31] and oxidative status [32].

The regulation of vascular reactivity by estrogen is mainly related to the maintenance of normal endothelial function [33]. Indeed, enhanced acetylcholine-induced vasodilation mediated by NO release in arteries isolated from estrogen-treated ovariectomized rabbits was one of the first evidence indicating the role of estrogen in vascular tone [34]. In endothelial cells, the modulation of NO bioavailability by estrogen has been extensively studied and is attributed to both genomic and non-genomic effects [35,36,37]. In addition to NO, the action of estrogen has also been implicated in the release of other endothelial-derived molecules such as prostacyclin [38] and angiotensin (Ang) 1–7 [39] and a decrease in endothelin-1 bioavailability [40] and Ang II receptor type 1 expression [41], thus reducing vasoconstriction and promoting vasodilation.

Besides their effect on vasomotor regulation, the anti-inflammatory responses induced by estrogen have been described in in vitro assays as well as in different vascular-injury models [42,43,44,45]. In this regard, estrogen reduces cell adhesion molecule expression in endothelial cells exposed to pro-inflammatory stimuli [46,47], and significantly decreases the cytokine-induced adhesion of monocytes to endothelium [48,49]. Moreover, the modulation of neutrophil chemotaxis [44] and leukocyte infiltration [45] by estradiol has been described in rat carotid arteries after acute injury. Estrogen treatment after rat carotid artery damage [50] also attenuates neointima formation by increasing endothelial cell growth and decreasing VSMC proliferation.

Estrogen also participates in the regulation of lipid accumulation in the vascular wall by modulating the plasma lipid profile and inhibiting the direct action of lipids on the vascular system. On the one hand, estrogen reduces the level of circulating cholesterol [51] and the rate of conversion of hepatic low-density lipoprotein (LDL) into bile acids [52] while on the other, it increases high-density lipoprotein (HDL) levels [53]. In addition, estrogen is associated with reduced lipid loading in human monocyte-derived macrophages [54] and VSMCs [55], preventing foam cell formation. Furthermore, estradiol exposure inhibits cellular permeability [56] and apoptosis [57] in LDL-exposed endothelial cells. Finally, estrogen attenuates the oxidative stress-mediated increase in LDL modifications, which accelerates lipid accumulation in arterial walls [58].

Although the antioxidant properties of steroids were first attributed to their phenolic structure [59], estrogen can also modulate antioxidant enzyme expression [60,61]. For instance, estradiol attenuates Ang II-induced superoxide production by increasing superoxide dismutase activity and protein expression in VSMCs [60] and endothelial cells [61]. Estradiol also reduces superoxide production by inhibiting nicotinamide adenine dinucleotide phosphate (NADPH) oxidase expression, thus reducing adhesion molecule and cytokine expression in VSMCs [62] and, in an experimental murine model of menopause, by reverting cyclooxygenase (COX) 2-dependent superoxide production in aortic tissue [63].

## 3. miRNA as Epigenetic Regulatory Mechanism

As previously described, classical regulation of physiological processes by estradiol includes estrogen signaling induced by direct and indirect target gene transcription. However, epigenetic mechanisms have recently emerged as another important source of gene expression regulation and are being widely studied. At the molecular level, epigenetics is based on three main pathways: (1) DNA methylation; (2) histone density, variants, and post-translational modifications; and (3) RNA-based mechanisms [64]. Together, these pathways are characterized by their ability to influence gene expression without changing the DNA sequence and many have been established as fundamental determinants of cardiovascular health and disease [65,66].

There is some evidence that epigenetic estrogen-regulation mechanisms are implicated in the regulation of cardiovascular function. For example, genes encoding ERs are more methylated (denoting the suppression of estrogenic activity) in atherosclerotic plaques compared to non-plaque regions in vascular tissues [67,68], thus suggesting that epigenetic ER inhibition plays an important role in atherosclerosis formation. On the other hand, histone modifications and chromatin remodeling also likely have estrogen-dependent effects on the vasculature [69,70]. Indeed, divergent estrogen-dependent gene expression in endothelial cells and VSMCs is linked to differential target-gene promoter histone acetylation [69]. Moreover, the vascular dysfunction prevented by estradiol is associated with histone 3 acetylation in a post-menopausal metabolic syndrome experimental model [70]. Finally, RNA-based epigenetic gene-expression regulatory mechanisms mediated by sequence-specific interactions have more recently been described and are our main focus in this review.

Regulatory non-coding RNA can be classified depending on the RNA length. Long non-coding RNA (lncRNA) is a heterogenic class of RNA that includes intergenic lncRNA, antisense transcripts, and enhancer RNA. All of them are described as non-protein-coding transcripts larger than 200 nucleotides (nt) so as to differentiate them from small non-coding RNAs [71]. These include microRNA (miRNA), small interfering RNA (siRNA), and Piwi-interacting RNA (piRNA), and are defined as small (20–30 nt) RNAs, which are associated with Argonaute (AGO) family proteins [72]. Moreover, a new class of non-coding RNAs derived from sequences located adjacent to miRNAs, termed miRNA offset RNA (moR), has been described [73]. Although moRs were first considered a by-product of miRNA biogenesis, recent studies have provided evidence that are biologically active and can alter gene expression to regulate cell proliferation in VSMCs [74].

**Figure 1:**
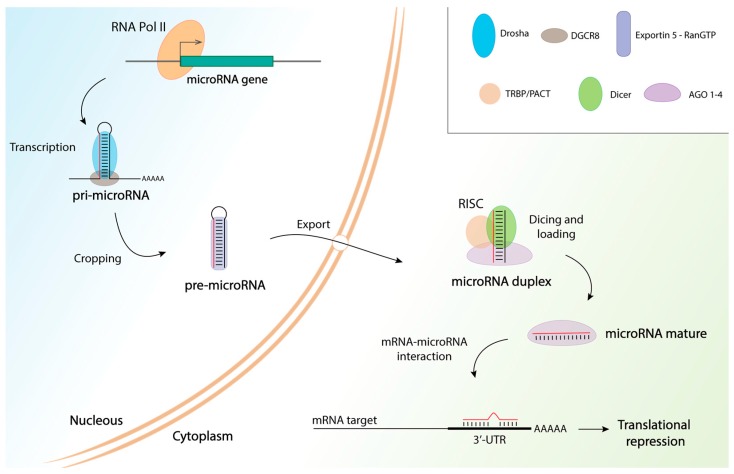
MicroRNA biosynthesis pathway. MicroRNAs (miRNAs) are transcribed by RNA polymerase II (Pol II) activity to generate the primary transcripts (pri-miRNAs). miRNA production is a two-step process involving nuclear cropping and cytosolic dicing processes. First, pri-miRNA cleavage is mediated by a processing complex comprising the RNase III, Drosha, and DiGeorge syndrome critical region 8 (DGCR8), which is also known as the microprocessor complex. This generates a hairpin-shaped pre-miRNA, which is recognized by nuclear exportin 5 and is exported to the cytoplasm where the RNase III, Dicer, cleaves pre-miRNA into 22-nucleotide miRNA duplexes. One strand from the cleavage products remains as a mature miRNA on the Argonaute (AGO) 1–4 proteins, whereas the other strand is degraded. Dicer, TAR RNA-binding protein (TRBP), protein kinase R-activating protein (PACT), and AGO 1–4 proteins mediate the assembly of the RISC (RNA-induced silencing complex). Finally, miRNAs guide translational repression by base-pairing with their target mRNAs, while AGO proteins recruit factors that induce this translational repression.

miRNAs about 20–22 nt long are the dominant class of small non-coding RNA in most tissues and are derived from nuclear transcripts with characteristic stem–loop structures (pri-miRNAs). The first step in miRNA biosynthesis is pri-miRNA cleavage, mediated by a processing complex comprising the RNase III Drosha and DiGeorge syndrome critical region 8 (DGCR8), also known as the microprocessor complex. Nuclear processing involves cropping the stem–loop to release a small hairpin-shaped RNA (pre-miRNA), which is then transported into the cytoplasm through exportin 5 where maturation can be completed. The second processing step is mediated by the RNase III, DICER1, which cleaves the pre-miRNA into 22-nt miRNA duplexes. Usually, one strand from the cleavage products remains as a mature miRNA due to a selective process that depends on thermodynamic stability. Finally, RNA generated is loaded into an AGO protein to form the effector RNA-induced silencing complex (RISC) along with other component such as TAR RNA-binding protein (TRBP) or protein kinase R-activating protein (PACT). miRNAs function as a guide by base pairing with their target messenger RNAs (mRNAs), while AGO proteins recruit factors that induce this translational repression; miRNA-binding sites are usually located at the 3′-untranslated region (UTR) of the target mRNA [75]. Figure 1 shows a schematic of the miRNA biosynthesis pathway along with most of the relevant implicated molecules.

Although no specific research has so far focused on the influence estrogen might exert on miRNA biosynthesis in vascular tissues, our group’s work on human endothelial cells treated with estradiol produced mRNA microarray data revealing the deregulation of key miRNA biosynthesis pathway genes [76]. Our data shows DGCR8 upregulation and DICER1 and AGO-2 downregulation in estradiol-treated cells (Table 1), suggesting that estrogen regulates endothelial miRNA production machinery.

In addition to data obtained in estradiol-treated endothelial cells, the relationship between estrogen action and miRNA biosynthesis has been extensively described in breast cancer samples, where differences in key miRNA-processing genes have been observed between ER+ and ER− breast cancer cells [77,78]. Specifically, the expression of DICER1, DGCR8, and DROSHA was higher, and that of AGO-2 lower, in ER+ breast tumors. In addition, of the miRNA processing genes this group studied, only DICER1 contains an ERα binding site in its regulatory region [79]. Indeed, miRNAs that are differentially expressed between ERα− and ERα+ breast cancer cells negatively control DICER1 expression [80], suggesting that a regulatory loop exists between ERs and miRNAs. In addition, other studies suggest that ERs interact with DROSHA to modulate its activity in breast cancer cells [81] and that a significant increase in Exportin-5 mRNA is induced in the mouse uterus by the action of estrogen [82].

Specific miRNAs target ERs and could therefore act as important ER-dependent gene expression modulators. Indeed, some estrogen-induced miRNAs such as miR-18a, miR-19b, and miR-20b target and regulate ERα expression, thus forming a negative feedback loop [83]. Other miRNAs, including miR-18a, miR-22, miR-206, and miR-221/222 have also been implicated in ERα targeting [84]. Finally, the only miRNAs identified as targeting ERβ [85] and GPER [86], respectively, are miR-92 and miR-424.

## 4. Vascular miRNA and Estrogen Action

The importance of miRNAs in vascular biology was first observed in 2005 by Yang et al., who described impaired vascular formation in DICER1 knockout mice [87]. In endothelial cells, DICER1 knockdown resulted in impaired proliferation and vessel formation, as well as altered expression of key proteins implicated in vascular tone regulation and angiogenesis, such as vascular endothelial growth factor receptor 2 (VEGFR), interleukin 8 (IL-8), and endothelial NO synthases (eNOS) [88,89], thus suggesting a role of miRNAs production in endothelial and vascular function.

Sex differences in miRNA expression have also been described in different physiological and pathological conditions [90,91], providing evidence for a role for sex hormones in miRNA regulation. Nevertheless, the relationship between sex-dependent miRNA expression and cardiovascular diseases has so far been little explored [90], although regulation of miRNA expression by estrogen was observed in different cell types and tissues [92]. In addition, the role of estrogen in the circulating miRNA profile has been described in both ovariectomized rats and postmenopausal women receiving hormone replacement treatments [93,94]; based on these results, different authors have proposed using these miRNA profiles as possible biomarkers for pathologies involving estrogen.

### 4.1. Estrogen-Dependent miRNA and Cardiovascular Function

Different studies have proposed that estrogen exerts its vascular protective effects, at least in part, via miRNA activity. For instance, the role of estrogen-induced miRNAs in heart tissue, VSMCs, and endothelial cells has been described; Table 2 summarizes the main miRNAs involved in the action of estrogen at cardiovascular level. Additionally, sex-dimorphic miRNA expression in heart tissue from males versus females has been noted, including for miR-222. As previously mentioned, this miRNA is involved in ERα regulation [84] and is implicated in modulating eNOS expression in cardiomyocytes by directly inhibiting the transcription factor ets-1 [95]. These results suggest that estrogen plays a role in regulating both the miRNA expression profile in cardiac tissues as well as the key molecules involved in cardiac function. In addition, miR-21, miR-24, miR-27a/b, and miR-106a/b were among the sex-specific miRNAs expressed via ERβ modulation in a murine model of pressure overload-induced cardiac fibrosis [96] and could help explain the differences in adaptation to pressure overload and vascular remodeling observed between women and men [97].

Important roles for miR-23a and miR-22 have also been described in cardiac function involving the action of estrogen. Specifically, miR-23a has regulatory regions containing ERα binding sites and plays a protective role in estrogen deficiency-induced cardiac gap-junction damage in rats [98]. The authors showed that estradiol inhibits miR-23-dependent downregulation of connexin 43 in a menopausal rat model, and provide new mechanisms of post-menopause-related arrhythmia [99]. In addition to its role in cardiac function, miR-23a levels also differ in males and females after cerebral ischemia and are related to accelerating apoptosis by regulating X-linked inhibitor of apoptosis (XIAP) expression and XIAP-caspase complex formation [100]. Thus, this evidence provides new insights into the molecular mechanisms underlying the sex-dependent responses observed following stroke [101]. Moreover, miR-22 provides estrogenic cardioprotection in female rats by controlling myocardial oxidative stress [102]. This same study also described a reciprocal feedback loop between ERα and miR-22, suggesting that estrogen action is closely regulated via post-transcriptional control of ERα expression. Similarly, the sex-specific regulation of miR-22 processing in muscle lipid metabolism has also recently been described and may contribute to understanding the well-described differences in muscle metabolism and body weight between males and females [103].

Considering vascular tissue, some studies show that VSMC proliferation is affected by miRNAs and highlight their potential as therapeutic agents in the treatment of proliferative cardiovascular diseases. In the case of mouse aorta, miR-203 contributes to the inhibition of VSMC proliferation because its upregulation is ER-dependent [104]. Estradiol induces miR-143 and miR-145 expression in pulmonary artery VSMCs via specific ER binding sites located in their promoter regions [105]. Moreover, estradiol-treated VSMCs secrete exosomes enriched with miR-143 and miR-145 which regulate VSMC-endothelium crosstalk in pulmonary arterial hypertension [105].

Focusing on the endothelium, microarrays were recently used to reveal that physiological (1 nmol/L) estradiol concentrations induce changes in the miRNA expression profile of endothelial cells [106]; among these, the miRNAs with the strongest differential expression were miR-30b-5p, miR-487a-5p, miR-4710, miR-501-3p, miR-378h, and miR-1244. Functional analysis using bioinformatic tools revealed that estradiol-modulated miRNAs were associated with key molecular pathways such as extracellular signaling from signal-regulated kinase/mitogen activated protein kinase (ERK/MAPK), integrins, and actin cytoskeleton signaling, which are important pathways in the regulation of vascular physiology in health and disease [106]. Additionally, most validated estradiol-regulated miRNAs were modulated by ERα, and to a lesser extent, by ERβ and GPER [106], thus lending weight to the idea that ERα plays a crucial role in estradiol-dependent effects on vascular tissues. On the other hand, estradiol is also implicated in the increased miR-126-3p expression observed in endothelial cells, resulting in increased cell migration, proliferation, and tube formation and decreased monocyte adhesion [107].

As previously described, estrogen plays a key role in modulating the immune system and this is probably the underlying cause of the sex differences observed in the inflammatory processes of atherosclerosis [108]. For instance, estradiol is involved in nuclear factor-kB (NF-kB) activity inhibition by regulating let-7a and miR-125b expression in stimulated macrophages [109]. Moreover, specific estradiol-regulated miRNAs—miR-146a and miR-223—have been described as key regulators of lipopolysaccharide-induced interferon-gamma (IFNγ) in lymphocytes [110]. Therefore, selective miRNA expression regulated by estrogen in immune cells could also be involved in the sex dimorphism observed in vascular diseases.

### 4.2. miRNA and Hormone Replacement Therapy

The use of HRT has recently been associated with the miRNA content of circulating exosomes in women [94]. In addition, the miRNA-mediated effects of this type of estrogenic therapy appear to improve the parameters of some disorders such as osteoporosis and sarcopenia and help to reduce the inflammation markers associated with these phenomena in postmenopausal women using HRT.

Although the relationship between estrogen levels and osteoporosis has been established for decades [113], changes in the miRNA expression profile in bone tissue from ovariectomy-induced osteoporotic mice and in postmenopausal women have recently been described [93,112]. Specifically, from among the miRNAs that are differentially expressed in estrogen-depleted mice, miR-127 and miR-136 negatively regulate bone mass [112], whereas miR-30b-5p may be a suitable serum biomarker for osteoporosis and osteopenia in postmenopausal women [93]. Moreover, suppressing the expression of miR-182 and miR-223, both implicated in regulating the insulin/insulin-like growth factor (IGF-1) pathway, in the skeletal muscle of postmenopausal women using HRT plays a central role in muscle mass regulation [111]. Therefore, the identification of estrogen-regulated miRNAs could be used as possible therapeutic targets to provide new insights into aging-related disorders such as sarcopenia. In addition, a study in monozygotic twin pairs revealed a relationship between changes in serum inflammatory markers and inflammatory-related miRNAs such as miR-21 and miR-146a, in postmenopausal women using HRT [114]. Thus, estrogen-sensitive miRNAs could be used as potential biomarkers for specific physiological deteriorations associated with female aging.

In another study, in premenopausal women and their monozygotic postmenopausal twins using estrogenic HRT, other circulating miRNAs included in exosomes, such as miR-148a-3p, miR-27-3p, miR-28-3p, miR-30a-5p, miR-106b-5p, and miR-126-5p were associated with serum estradiol levels [94]. miR-148a is related to regulation of plasma LDL/HDL ratio by directly regulating hepatic LDL receptor (LDLR) [115]. This effect could be related to the previously demonstrated effects of estrogen on circulating cholesterol levels as estrogen is implicated in the reduction of circulating cholesterol by increasing LDLR expression [116]. Another estrogen-related miRNA, miR-27, is also implicated in LDLR expression without producing changes in plasma cholesterol levels [117]; this miRNA is also related to angiogenic processes [89] and was recently suggested as a biomarker for stenotic progression in asymptomatic carotid stenosis [118]. In this regard, there are sex-related differences in patients with this pathology [119] that may be partly related to the role of estrogen-regulated miRNAs. Therefore, a better understanding of the mechanisms underlying these processes could improve new sex-specific therapeutic approaches.

MiR-106b-5p decreases tumor necrosis factor (TNF) α-induced apoptosis by repressing phosphatase and tensin homolog (PTEN)-caspase activity in vascular endothelial cells [120]. Moreover, these effects correlate with the repressive effects that estrogen have on PTEN and apoptosis [121,122]. miR-126-5p is required to produce correct vascular integrity and is key in angiogenic processes [123,124] and also decreases leukocyte-endothelium interactions by suppressing vascular cell adhesion molecule (VCAM)-1 [125]. In line with the aforementioned studies, miR-126-5p is among the estradiol-regulated miRNAs present in endothelial cells [107]. Therefore, the estradiol-sensitive miRNAs described could provide insight into the mechanisms by which estrogen modulates important endothelial processes such as apoptosis or angiogenesis to provide correct vascular physiology.

## 5. Conclusions

The differences observed in cardiovascular diseases between the sexes attribute a protective role to estrogen, which is mediated through the regulation of transcription processes and, in turn, cellular physiology. Indeed, sex-biased gene expression in the cardiovascular system and mediated by estrogen has already been reported. It is estimated that miRNAs regulate the expression of approximately 30% of all protein-coding genes in mammals, implying their importance in correctly functioning human physiology, including that of the cardiovascular system. However, although there is increasing evidence to establish epigenetic mechanisms, including miRNAs, as crucial regulators of vascular function, the role of miRNAs in estrogen-mediated vascular functions must still be elucidated. Therefore, future research focused on characterizing the role of specific estradiol-mediated miRNAs involved in vascular function will be required to provide new knowledge about how the levels of sex hormones can contribute to sex-related differences in cardiovascular diseases.

## Figures and Tables

**Table 1:** Microarray expression data for key miRNA biosynthesis pathway molecules. mRNA expression data were obtained from previously published mRNA microarray data obtained for human umbilical vein endothelial cells (HUVECs) treated with 1 nmol/L estradiol for 24 h. The probe set ID, gene symbol, official full name, *p*-value, and fold change are shown. These mRNA microarray data are deposited in NCBI’s Gene Expression Omnibus (http://www.ncbi.nlm.nih.gov/geo), accessible through GEO series accession number GSE16683.

Probe Set ID	Symbol	Official Full Name	Fold Change	*p* Value
218269_at	DROSHA	drosha, ribonuclease type III	−1.117	0.586
64474_g_at	DGCR8	DiGeorge syndrome critical region gene 8	2.376	0.016
223056_s_at	XPO5	exportin 5	1.514	0.259
213229_at	DICER1	dicer 1, ribonuclease type III	−1.979	0.012
225569_at	AGO-2	argonaute-2	−1.290	0.002

**Table 2:** miRNA-dependent estrogen actions. Focusing on the role of estrogen in cardiovascular system and in HRT, estrogen-dependent effect and its associated estrogen-related miRNA are shown.

Estrogen Action	miRNA	References
Sex differences in heart	miR-1 miR-106b miR-720 miR-29b miR-144 miR-34b-5p miR-205 miR-222	[95]
Sex differences in cardiac fibrosis	miR-21 miR-24 miR-27a/b miR-106a/b	[96]
Cardiac gap junction regulation	miR-23a	[98]
Regulation of oxidative stress in the myocardium	miR-22	[102]
Inhibition of VSMC proliferation	miR-203	[104]
VSMC and endothelial cell communication	miR-143 miR-145	[105]
Endothelial cell proliferation	miR-126-3p	[107]
miRNA expression profile in estradiol-treated endothelial cells	miR-30b-5p miR487a-5p miR-4710 miR-501-3p miR-378h miR-1244	[106]
Regulation of NF-kB pathway in macrophages	let-7a and miR-125b	[109]
Regulation of IFNγ released in lymphocytes	miR-146a miR-223	[110]
Regulation of Insulin/IGF-1 pathway in skeletal muscle	miR-182 and miR-223	[111]
Circulating Inflammation markers	miR-21 miR-146a	[105]
Negative regulation of bone mass.	miR-127 and miR-136	[112]
Serum biomarker in osteoporosis	miR-30b-5p	[93]
Circulating miRNA	miR-106-5p miR-148a-3p miR-27-3p miR-126-5p miR-28-3p miR-30a-5p	[94]
